# The Many Faces of Neurological Neonatal Herpes Simplex Virus Infection

**DOI:** 10.7759/cureus.41580

**Published:** 2023-07-08

**Authors:** Fadi Shahoud, Mobeen H Rathore, Chetan C Shah, Rana Alissa

**Affiliations:** 1 Pediatrics/Infectious Diseases, University of Florida College of Medicine, Jacksonville, USA; 2 Pediatric Radiology, Nemours Children's Health System, Jacksonville, USA; 3 Pediatrics, University of Florida College of Medicine, Jacksonville, USA

**Keywords:** hsv pcr, neonatal sepsis, neonatal hsv infection, hsv encephalitis, herpes simplex virus infection

## Abstract

This case series explores the various manifestations of central nervous system (CNS) involvement in neonatal herpes simplex virus (HSV) infection and highlights the challenges involved in their diagnosis and treatment. Neonatal HSV infection is a rare but serious condition that can have significant neurological consequences. The article presents three cases of neonatal HSV infection, all involving the CNS, each characterized by distinct clinical features and outcomes. Case 1 describes a three-week-old male with severe HSV meningoencephalitis resulting in poor response to treatment and death. Cases 2 and 3 describe younger neonates who presented early in the disease course with disseminated infection and skin, eye, and mouth (SEM) lesions. Although both patients had CNS involvement, their outcomes were remarkably favorable.

The wide range of clinical presentations of CNS manifestations in neonatal HSV infection, ranging from nonspecific to evident neurological symptoms, underscores the need for a high index of suspicion and comprehensive evaluation to ensure early diagnosis and appropriate treatment. However, it also notes that even with timely treatment, some cases may still have a poor prognosis.

## Introduction

Neonatal herpes simplex virus (HSV) infection is a rare yet serious and potentially life-threatening condition transmitted from mother to newborn during childbirth and primarily affects newborn infants within the first month of life. Several risk factors have been identified, including the presence of maternal genital lesions or active genital herpes outbreaks at the time of delivery, a lack of maternal antibodies against HSV, and the prolonged rupture of membranes. Other factors that increase the risk of neonatal herpes infection include primary maternal infection during late pregnancy and invasive obstetric procedures such as the use of fetal scalp electrodes and other instrumentation [1].

Neonatal HSV infection is categorized into three types: the first type is known as skin, eye, and mouth (SEM) disease, characterized by herpetic lesions in these specific areas. The second type is central nervous system (CNS) disease, which may occur with or without SEM disease manifestations. Lastly, there is disseminated disease, which can affect multiple organ systems and may or may not be accompanied by CNS disease or SEM disease manifestations [2].

CNS neonatal HSV infection can have a significant neurological impact leading to a wide range of clinical manifestations including seizures, temperature instability, lethargy, poor feeding, irritability, altered mental status, and focal neurological deficits. The timely identification and management of neonatal HSV infection are important in preventing long-term complications and improving outcomes for affected infants. However, even when treated appropriately and in a timely manner, there could still be a poor outcome. This article aims to explore the diverse presentation of CNS manifestations in neonatal HSV infections and highlight the challenges involved in their diagnosis and treatment.

## Case presentation

Case 1

A three-week-old male, born at 37 weeks of gestation via spontaneous vaginal delivery, presented to the emergency department (ED) with two days of excessive sleepiness and decreased feeding. The mother denied fever, runny nose, diarrhea, vomiting, seizure-like activity, or sick contacts. Maternal history was significant for group B streptococci (GBS) vaginal colonization, which was treated appropriately with intrapartum ampicillin. The mother also had a history of HSV genital infection; however, there were no active lesions during pregnancy, labor, or delivery. Labor and delivery were uneventful. On examination, rectal temperature was 35.7°C, blood pressure (BP) was 64/43 mm Hg, heart rate was 134 beats per minute, respiratory rate was 36 breaths per minute, and oxygen saturation (SpO_2_) on pulse oximetry was 97% in room air. He was obtunded and unresponsive to painful stimuli. There was no bulging of the anterior fontanelle. The pupils were symmetric but sluggishly reactive. There were no hepatomegaly or mucocutaneous lesions.

A sepsis evaluation was performed, including blood and urine cultures, followed by the administration of empiric treatment with intravenous (IV) ampicillin, gentamicin, and acyclovir. His complete blood count (CBC) on a peripheral smear demonstrated a white blood cell (WBC) count of 4,100/mm^3^ with 50% neutrophils, 36% lymphocytes, 11% monocytes, 2% eosinophils, and 1% basophils; hemoglobin of 15.6 g/dL; and platelet of 308,000/mm^3^. His procalcitonin was 0.09 ng/mL (normal range {NR}: 0.00-0.50 ng/mL), aspartate transferase (AST) was 27 IU/L (NR: 14-54), and alanine aminotransferase (ALT) was 13 IU/L (NR: 10.00-60.00). Chest radiograph was normal.

**Figure 1:**
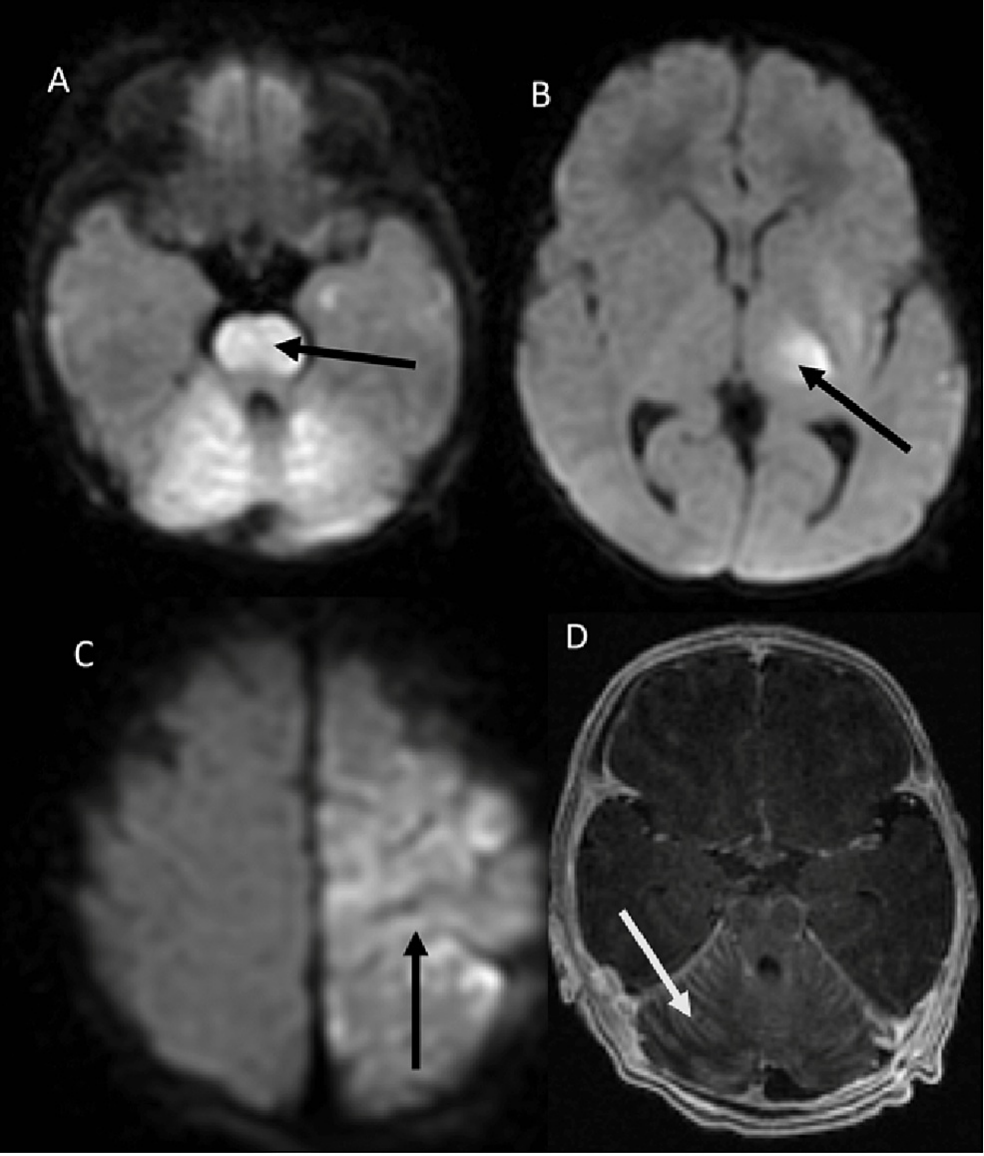
Diffusion-weighted axial MRI images of the brain (A, B, and C) show multiple areas of acute infarcts including the brainstem (arrow in A), left thalamus (arrow in B), and left motor cortex (arrow in C). Contrast-enhanced T1-weighted axial MRI image of the brain (D) at the level of the cerebellum shows extensive leptomeningeal enhancement (arrow in D).

A computed tomography (CT) scan of the head without contrast enhancement revealed no intracranial hemorrhage. The patient subsequently developed apneic events requiring mechanical ventilation. On the first hospital day, an electroencephalogram (EEG) recorded abnormal electrical activity suggestive of subclinical seizure activity. Phenobarbital and fosphenytoin were started. Brain MRI with contrast enhancement revealed non-hemorrhagic infarcts involving the bilateral cerebellar hemispheres, brainstem, left thalamus, left posterior limb of the internal capsule, corona radiate, and left frontal and parietal lobes along with diffuse leptomeningeal enhancement (Figure 1).

Cerebrospinal fluid (CSF) examination revealed a white cell count of 34/mm^3^ (45% lymphocyte, 54% monocyte, and 1% neutrophils), red blood cells (RBC) of 5/mm^3^, a protein level of 174 mg/dL (NR: 10-65), and a glucose level of 46 mg/dL (NR: 50-75) (blood glucose of 90 mg/dL). A CSF and blood HSV-1/2 polymerase chain reaction (PCR) was positive for HSV-2. HSV PCR from the conjunctival sac, rectum, and oropharynx/nasopharynx was negative. CSF enterovirus PCR was also negative. Blood, urine, and CSF bacterial cultures remained sterile. Ampicillin and gentamicin were discontinued, and the patient continued treatment with acyclovir 60 mg/kg per day in three divided doses for HSV-2 meningoencephalitis.

**Figure 2:**
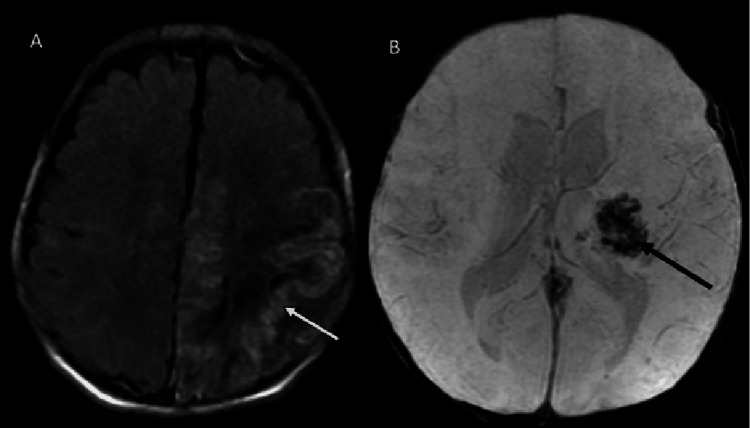
Axial fluid-attenuated inversion recovery (FLAIR) MRI image (A) on the follow-up MRI performed after 10 days shows laminar necrosis (arrow) and encephalomalacia in left frontal lobe. Susceptibility-weighted axial MRI image (B) shows hemosiderin from hemorrhage (arrow) in the left thalamus.

After two weeks of IV acyclovir, the infant still had no clinical improvement and poor respiratory drive and failed multiple extubation attempts. Repeat MRI showed the development of laminar necrosis encephalomalacia with hemorrhagic products noted at the site of infarcts (Figure 2). Life support was withdrawn, and the patient was provided compassionate supportive care to allow natural death.

Case 2

A six-day-old female, born at term via spontaneous vaginal delivery, presented to the ED with one day of fever, decreased activity, and poor feeding. The mother denied a history of cough, runny nose, diarrhea, vomiting, or skin rash. There was no maternal history of HSV. Pregnancy, labor, and delivery were uncomplicated. On examination, rectal temperature was 38.6°C, BP was 69/47 mm Hg, heart rate was 191 beats per minute, respiratory rate was 50 breaths per minute, and SpO_2_ on pulse oximetry in room air was 99%. She was lethargic and irritable but was easily consoled. The anterior fontanelle was not bulging. There were no hepatomegaly or mucocutaneous lesions. The rest of the examination was unremarkable.

A sepsis evaluation was performed, including blood, urine, and CSF cultures. She was empirically started on IV ampicillin, gentamicin, and acyclovir. Chest radiograph was normal. CBC on a peripheral smear revealed a WBC count of 5,800/mm^3^ with 52% neutrophils, 37% lymphocytes, 8% monocytes, 2% eosinophils, and 1% basophils; hemoglobin of 16.4 g/dL; and platelet of 136,000/mm^3^. The initial ALT was 85 U/L, and AST was 342 U/L. The prothrombin time (PT) was 16.4 seconds (NR: 11.8-15), and the partial thromboplastin time (PTT) was 45 seconds (NR: 22.9-37.8). CSF analysis revealed a WBC count of 1/mm^3^ with 64% lymphocyte, 27% monocyte, and 9% histiocytes; a RBC of 1/mm^3^; a protein level of 60 mg/dL; and a glucose level of 66 mg/dL (blood glucose of 101 mg/dL). A CSF HSV-1/2 PCR was positive for HSV-2, suggesting disseminated HSV disease with CNS involvement. Surface and blood HSV PCR tests were not done. Ampicillin and gentamicin were discontinued due to sterile bacterial cultures, and the patient continued treatment with acyclovir 60 mg/kg per day in three divided doses.

**Figure 3:**
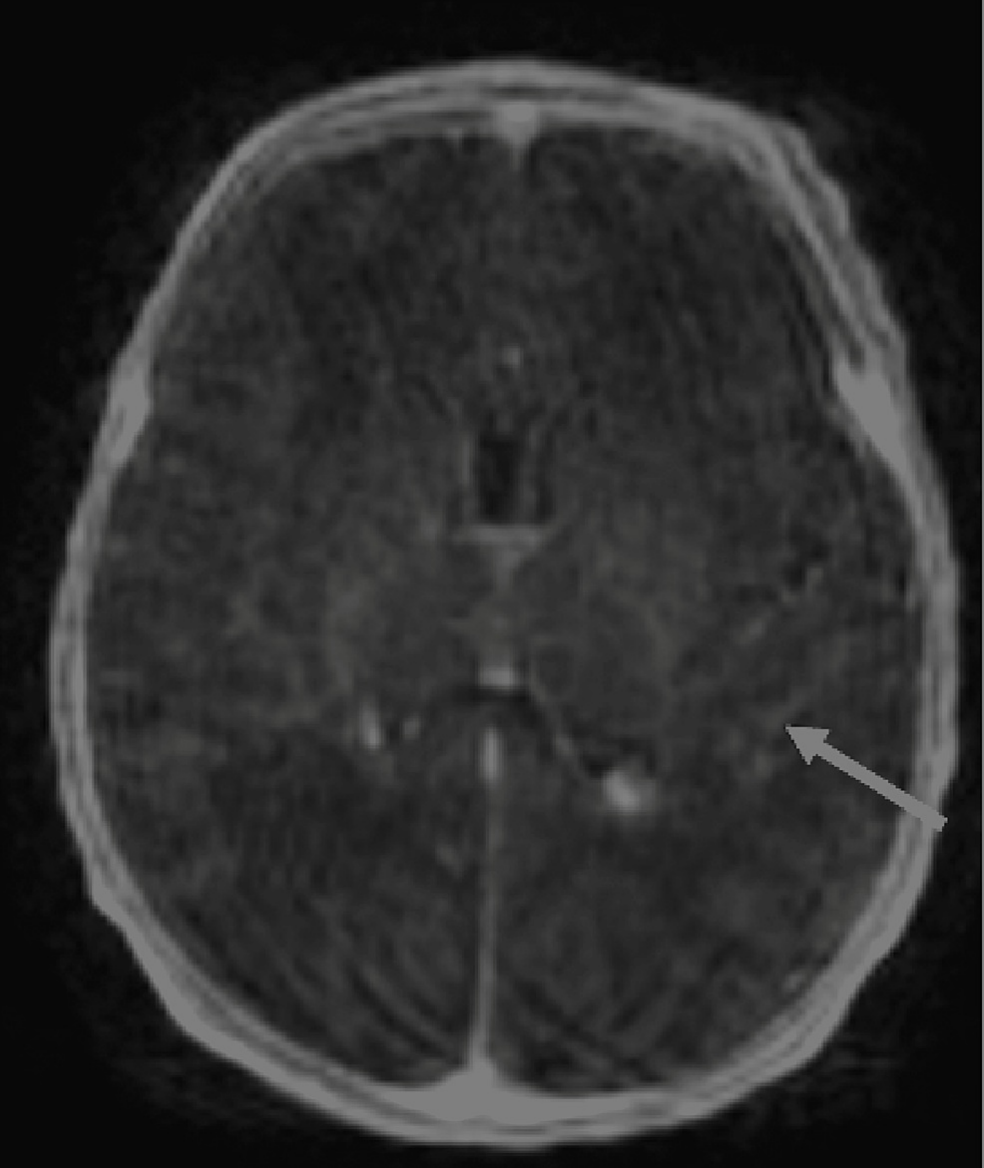
Contrast-enhanced T1-weighted axial MRI image of the brain shows leptomeningeal enhancement (arrow).

During the second and third days of her hospital stay, the infant continued to be febrile and irritable with poor activity and decreased interest in feeding. Repeat liver enzyme levels showed a rising ALT of 878 IU/L and AST of 3,495 IU/L. A contrast-enhanced MRI of the brain revealed diffuse leptomeningeal enhancement. Otherwise, there was no visualized restricted diffusion or evidence of acute intracranial hemorrhage (Figure 3).

On hospital day 4, she demonstrated gradual clinical improvement. Liver enzymes dramatically declined and returned to normal levels on hospital day 10. After completing 21 days of IV acyclovir treatment, a repeat CSF HSV PCR was negative. She was discharged with a planned six-month course of oral acyclovir suppression at a dose of 300 mg/m^2^/dose, administered three times daily. During the follow-up visit at the conclusion of therapy, the patient was developmentally normal and achieved age-appropriate milestones, exhibiting appropriate growth, cognitive abilities, and motor skills.

Case 3

A five-day-old male infant, born at term via spontaneous vaginal delivery, presented to the ED with a two-day history of vesicular lesions on the scalp. He was initially treated with topical mupirocin for presumed bacterial involvement, but the scalp lesions did not improve, and new vesicular lesions appeared. The mother denied fever, lethargy, or decreased feeding. Maternal history was negative for HSV infection or active genital lesions during pregnancy, labor, and delivery. She had a positive GBS screen on a vaginal swab at delivery and was treated with intrapartum ampicillin. Otherwise, pregnancy, labor, and delivery were uneventful. There was no history of fetal scalp electrode use or herpes lesions in close contacts with the baby. On examination, rectal temperature was 37.8°C, BP was 72/54 mm Hg, heart rate was 155 beats per minute, respiratory rate was 40 breaths per minute, and SpO_2_ on pulse oximetry was 100%. There were three vesicles with an erythematous base on his scalp. There was no hepatomegaly, and the anterior fontanelle was not bulging. The rest of the examination was unremarkable.

**Figure 4:**
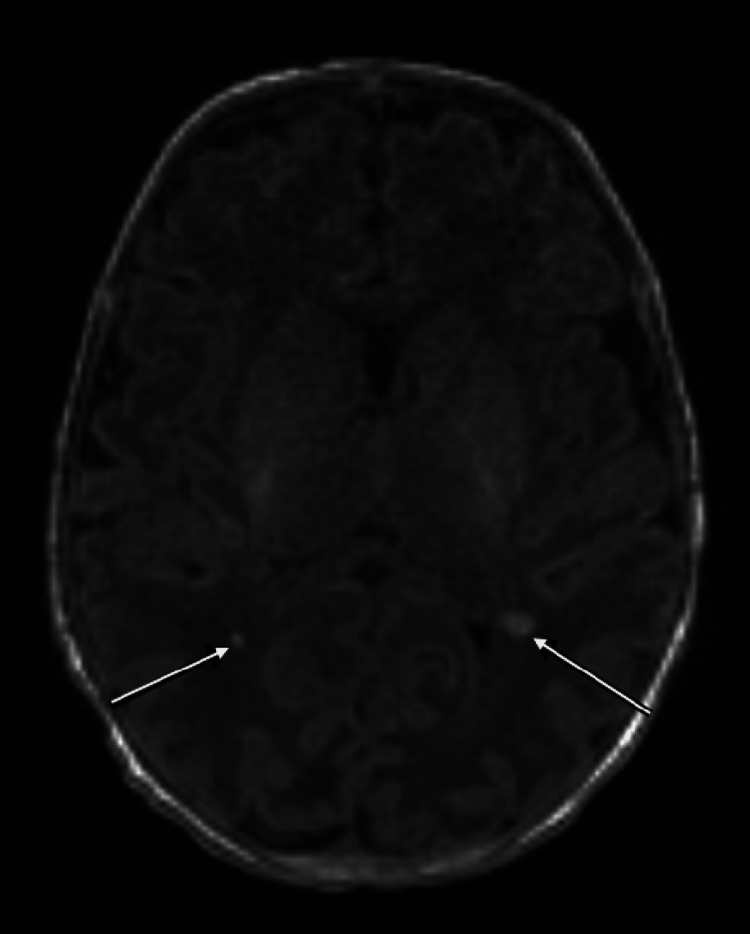
T1-weighted axial MRI image of the brain without the administration of intravenous contrast agent shows foci of hemorrhage in parietal white matter bilaterally (arrows).

The patient underwent a sepsis evaluation, including blood, urine, and CSF cultures. A swab from the scalp lesion was sent only for bacterial culture. The infant was empirically started on IV ampicillin, gentamicin, and acyclovir. Chest radiograph was normal. CBC on a peripheral smear revealed a WBC count of 8,300/mm^3^ with 50% neutrophils, 42% lymphocytes, and 8% monocytes; hemoglobin of 17.1 g/dL; and platelet of 147,000/mm^3^. ALT was 25 U/L, and AST was 45 U/L. CSF analysis revealed a white cell count of 3/mm^3^ with 60% lymphocyte, 35% monocyte, 4% neutrophils, and 1% eosinophils; red cells of 1,980/mm^3^; a protein level of 99 mg/dL; and a glucose level of 46 mg/dL (blood glucose of 70 mg/dL). An CSF HSV-1/2 PCR was positive for HSV-1. An HSV PCR test was not performed on scalp lesions and blood. An MRI of the head with/without contrast enhancement revealed small punctate areas of hemorrhage in the parietal white matter (Figure 4).

Ampicillin and gentamicin were discontinued due to sterile cultures, and the patient continued treatment with acyclovir 60 mg/kg per day in three divided doses for 21 days. He had an uneventful course of treatment and clinically improved. A repeat CSF HSV PCR at the end of treatment was negative. The patient was discharged with a planned six-month course of oral acyclovir suppressive therapy at 300 mg/m^2^/dose, administered three times daily. The patient was lost to follow-up.

## Discussion

The diagnosis of neonatal herpes infection can be challenging, as symptoms can be nonspecific and overlap with other conditions. A combination of clinical presentation, imaging studies such as MRI, and the detection of HSV DNA in CSF using PCR testing can provide a rapid and accurate diagnosis of HSV CNS infection [3]. This case series shows the varied clinical presentations and outcomes of neonatal HSV infection. Case 1 highlights the severity of HSV meningoencephalitis and emphasizes the possibility of rapid progression to a complicated illness that can be fatal. This patient was seen at three weeks of age. However, the CNS HSV infection probably started and was present much earlier while the patient remained asymptomatic. This is particularly an issue in a neonate who presents with purely CNS HSV infection without dissemination or SEM lesions. SEM lesions when present are often seen earlier in the course of neonatal HSV disease. As a result, SEM disease receives earlier evaluation and treatment for neonatal HSV even before the clinical evidence of CNS disease. This was the clinical scenario in cases 2 and 3 where the patients presented earlier in the disease course with disseminated and SEM diseases. Although both these cases had CNS involvement, the outcomes were drastically different. All three neonates had CNS HSV disease, and although the outcome in the two cases was favorable, this is not always the case. Many cases of neonatal CNS HSV infection even if treated early and appropriately may still have a poor outcome. Although the early treatment of CNS neonatal HSV infection does not guarantee a favorable outcome, late treatment is often associated with poor outcome. It is important to point out that late treatment is not delayed treatment since the neonate presents later in the disease course often with the manifestation of CNS disease. This was the scenario in the first case reported here. The initial symptoms of HSV CNS infection can be subtle and nonspecific such as fever, poor feeding, lethargy, fussiness, and shortness of breath, which may mimic other neonatal diseases and make diagnosis challenging [4]. The presence of vesicular eruptions on the skin or mucous membranes is highly suggestive of HSV infection and necessitates comprehensive evaluation to exclude disseminated HSV and the involvement of the CNS [5].

Approximately 30% of neonates with HSV infection present with central nervous system (CNS) disease, either with or without SEM manifestations. Additionally, up to 60% of neonates with disseminated HSV infection experience CNS involvement. The most common neurological manifestation of neonatal herpes infection is HSV encephalitis (HSE), which is commonly characterized by seizures (67%), obtundation (67%), fever/hypothermia (56%), bulging fontanelle, irritability, and abnormal neurological examination findings such as hypotonia or hypertonia. Infants with severe cases may develop apnea, coma, or paralysis [6,7].

Neonates with HSV encephalitis typically have mononuclear pleocytosis on presentation, along with depressed CSF glucose and mild to moderately elevated CSF protein [8,9]. Notably, these CSF findings were also observed in the patient with severe encephalitis in case 1, as opposed to cases 2 and 3, where the initial presentation lacked significant neurological symptoms and demonstrated a relatively normal CSF white cell count, mildly elevated protein levels, and normal glucose levels. This disparity in CSF findings could potentially indicate an early diagnosis in the latter two patients.

Neonates with severe HSV encephalitis commonly have abnormal neuroimaging findings. Cortical lesions are identified as the primary findings on diffusion-weighted imaging (DWI) during the early course. The presence of bilateral deep cerebral lesions, specifically involving the basal ganglia, internal capsules, and thalamus, was strongly associated with poor neurological outcomes [10].

The neurological outcomes of neonatal herpes infection can be variable and unpredictable and depend on various factors, including the severity of the initial symptoms and the time to treatment. Despite effective antiviral treatment, neonates with severe HSV encephalitis may have a poor prognosis, with up to 6% mortality and long-term neurodevelopmental deficits including developmental delay, intellectual disability, cerebral palsy, and epilepsy in up to 30%-70% of survivors [3,11,12]. Early intervention and ongoing management, including physical therapy, speech therapy, and educational support, can help minimize the impact of these deficits.

The important role of acyclovir in the management of neonatal herpes infection is supported by several studies [13,14]. The increased use of acyclovir in neonates being evaluated for sepsis could have remarkable benefits in neonates ultimately diagnosed with HSV infection [15-17]. Intravenous acyclovir at a dosage of 20 mg/kg every eight hours for at least 21 days is recommended to treat disseminated and CNS neonatal HSV infections. Neonates with CNS involvement should undergo a repeat lumbar puncture toward the end of therapy to confirm a negative CSF HSV PCR. If HSV PCR is positive, intravenous acyclovir should be continued for an additional seven days [18]. While acyclovir is generally well tolerated, there may be associated adverse effects, including kidney injury, which can occur if the neonate becomes dehydrated and crystallization happens in the renal tubules. It is important to ensure proper hydration to minimize this risk. In rare cases of extravasation, acyclovir may lead to phlebitis and potential damage to surrounding tissue. Additionally, reversible neutropenia, which is dependent on the dosage, can occur as a side effect of acyclovir. Foscarnet and cidofovir have been occasionally used as an alternative treatment in sporadic cases of acyclovir-resistant neonatal herpes infection. However, these two agents have not been extensively studied in neonatal populations, and both carry a significant risk of nephrotoxicity [18].

Several studies have concluded that a six-month course of acyclovir suppressive therapy in neonates with HSV infection is associated with a significant reduction in mortality, neurological impairment, and the recurrence of HSV infection [19,20].

## Conclusions

Neonatal CNS HSV infection can present with a wide range of neurological symptoms and disease courses, ranging from relatively benign meningitis to severe, life-threatening, and even fatal encephalitis/meningoencephalitis. Early diagnosis and prompt treatment with acyclovir are critical to improving outcomes in affected neonates but are not a guarantee. Clinicians should maintain a high index of suspicion for neonatal HSV infection in at-risk neonates with unexplained neurological or nonspecific symptoms, and treatment should be initiated immediately in suspected cases. It is appropriate to initiate empiric acyclovir therapy when HSV is a consideration. It is however important that appropriate evaluation for neonatal HSV including any skin or mucosal lesion, conjunctival sac, oropharynx/nasopharynx, blood, and CSF HSV PCR be performed preferably prior to starting acyclovir therapy.
